# Visual Colorimetric Sensing of the Animal-Derived Food Freshness by Juglone-Loaded Agarose Hydrogel

**DOI:** 10.3390/foods14142505

**Published:** 2025-07-17

**Authors:** Lanjing Wang, Weiyi Yan, Aijun Li, Huayin Zhang, Qian Xu

**Affiliations:** Key Laboratory of Environmental Medicine Engineering, Ministry of Education, School of Public Health, Southeast University, Nanjing 210009, China; wlj345369@163.com (L.W.); 220244293@seu.edu.cn (W.Y.); a2001jun@163.com (A.L.)

**Keywords:** juglone, colorimetric sensing, animal-derived food, freshness, dynamic monitoring

## Abstract

The visual colorimetric sensing of total volatile basic nitrogen (TVB-N) allows for convenient dynamic monitoring of animal-derived food freshness to ensure food safety. The agarose hydrogel loaded with the natural dye juglone (Jug@AG) prepared in this study exhibits visible multicolor changes from yellow to grayish-yellow and then to brownish with increasing TVB-N gas concentration, achieving sensitive detection of TVB-N gas at concentrations as low as 0.05 mg/dm^3^ within 8 min. The minimum observable amounts of TVB-N in spiked pork and fish samples are 8.43 mg/100 g and 8.27 mg/100 g, respectively, indicating that the Jug@AG hydrogel possesses sensitive colorimetric sensing capability in practical applications. The Jug@AG hydrogel also shows significant changes in color difference value (∆C) under both room temperature (25 °C) and cold storage (4 °C) conditions, with the changing trends of ∆C showing consistency with the measured TVB-N and total viable counts (TVC) during the transition of pork and fish samples from freshness to early spoilage and then to spoilage. The results indicate that the Jug@AG hydrogel can be used as a colorimetric sensor to achieve real-time dynamic freshness monitoring of animal-derived food.

## 1. Introduction

Total volatile basic nitrogen (TVB-N) is predominantly composed of ammonia along with other alkaline nitrogen-containing gases such as trimethylamine, dimethylamine, triethylamine, and propylamine [1]. As one of the main reference indicators for the freshness of animal-derived food [2], the European Union (EU) stipulates that the TVB-N content in fresh aquatic products must not exceed 25 mg/100 g [3]. The total viable counts (TVC) of bacteria such as *Salmonella*, *Escherichia coli*, and *Listeria* are also used in combination with TVB-N content to accurately determine freshness, and the food is deemed spoiled and unsafe when TVC exceeds 1 × 10^7^ CFU/g [4,5], which is equivalent to 7 in terms of base-10 logarithm, i.e., 7 lg (CFU/g). The National Food Safety Standards of China specify that the TVB-N content must not exceed 15 mg/100 g, 20 mg/100 g, and 30 mg/100 g in fresh livestock and poultry meat, freshwater fish and shrimp, and marine fish and shrimp, respectively [6,7]. Previous studies have further subdivided this standard and proposed that the TVB-N content in livestock and poultry, as well as freshwater fish and shrimp with optimal freshness, is less than 10 mg/100 g and 15 mg/100 g, respectively. The animal-derived food is classified as having moderate freshness when TVB-N content ranges from 10–15 mg/100 g in livestock and poultry meats or 15–20 mg/100 g in freshwater fish and shrimp [8,9], suggesting that these products are in the early spoilage stage and should be consumed with caution to prevent foodborne illnesses [10]. Therefore, real-time monitoring of TVB-N in animal-derived food during production, circulation, and consumption enables timely detection of early spoilage and serves as an effective safeguard for consumer health. Solid-phase colorimetric sensors facilitate visual detection of volatilized TVB-N during the spoilage of animal-derived foods [11]. Dyes are commonly used as probes in gas sensing systems, and the freshness of food can be determined by observing the color change of the sensor with the naked eye, which provides convenience for real-time monitoring [12]. Currently, the commonly used dyes include synthetic dyes such as bromocresol purple [8], phenol red [13], bromothymol blue [14], and azo dyes [15], as well as natural dyes such as anthocyanins [16,17,18,19], alizarin [20], and quercetin [21]. The TVB-N produced during the spoilage of animal-derived food is adsorbed by the hydrophilic carrier. The ammonia combines with water in the carrier to form ammonium hydroxide (NH_3_·H_2_O), which partially ionizes to NH_4_^+^ and OH^−^. Other nitrogenous compounds with lone-pair electrons on nitrogen atoms can accept the protons produced by the ionization of water molecules in the carrier to form ammonium ions. In the resulting alkaline environment, phenolic hydroxyl groups, carboxylic acid groups, and sulfonic acid groups within dye molecules undergo deprotonation, generating dye ions with colors distinct from those in their neutral forms. The deprotonation-induced color change causes visible color variations, enabling visual detection of food spoilage [22].

A hydrogel is a three-dimensional network gel formed from hydrophilic polymers rich in polar functional groups such as –OH, –COOH, and –NH_2_. These polymers endow hydrogels with the ability to absorb and retain large quantities of water, to effectively enrich TVB-N gases and to provide an alkaline environment for the structural change of the above dyes [23]. Their non-rigid and porous structural characteristics not only effectively reduce the steric hindrance effect, but also increase the contact area with gas molecules, facilitating colorimetric response and improving the sensing speed [24,25]. Hydrogels are excellent carriers for constructing TVB-N gas colorimetric sensors, among which agarose hydrogels have been employed in several cases [15,18,19,26]. Diana et al. prepared an agarose hydrogel encapsulated with a novel azo dye 3-hydroxy-4-((5-(2-hydroxy-4-nitrophenylazo)thiophen-2-imino)methyl)phenyl-4-(hexyloxy)benzoate, and the hydrogel exhibited a naked-eye visible color change from yellow to purple when exposed to TVB-N volatilized from chicken (pH 6.5) [15]. He et al. developed a pH-responsive absorbent pad using anthocyanin dye as a probe immobilized on polyvinyl alcohol (PVA)/agarose substrate. As the TVB-N content in pork progressed from early spoilage (10 mg/100 g) to complete spoilage (15 mg/100 g), the pad exhibited naked-eye-detectable color evolution from red through gray to green, enabling real-time dynamic monitoring of pork freshness [18].Zhai et al. fabricated a double-gel-layer colorimetric sensor by coating purple sweet potato anthocyanin agarose hydrogel with an oleogel composed of sunflower oil, beeswax, and monooleic glyceride. This sensor demonstrated visible color variation detectable with the naked eye upon exposure to trimethylamine gas with a concentration of 250 μmol/dm^3^ within 20 min. When TVB-N content in beef exceeded the spoilage threshold of 15 mg/100 g, a distinct color transition was observed, indicating freshness deterioration [19]. The application potential of solid-phase sensors based on dye-loaded hydrogels for colorimetric detection of TVB-N gas has been widely recognized by researchers.

Juglone, chemically known as 5-hydroxy-1,4-naphthoquinone, is a phenolic organic compound naturally found in the immature hulls of *Juglans regia* (common walnut) and its congener *Juglans nigra* (black walnut) within the Juglandaceae family. Juglone typically exists as yellow crystals or a fine powder. It has low solubility in water but demonstrates high solubility in various organic solvents such as acetone, benzene, chloroform, diethyl ether, and ethanol [27]. The presence of a hydroxyl group within its molecular structure confers weak acid properties, enabling reactions with strong bases. This acid-base characteristic significantly influences its chemical reactivity and biological activity [27]. Studies have shown that juglone exhibits pH-dependent color variations [28], indicating its potential as a sensing probe for TVB-N monitoring. Currently, Juglone is primarily used in the development of anti-cancer, anti-infective, and neuroprotective drugs [27], as well as in hair dye formulations and synthetic fiber coloration processes [29]. These diverse functionalities highlight its promising yet underexplored potential in colorimetric sensing applications.

In this work, a juglone-loaded agarose hydrogel (Jug@AG) was prepared and used as a colorimetric sensor for dynamic monitoring of the freshness of animal-derived food. After evaluating the colorimetric sensing feasibility of Jug@AG hydrogel toward five typical TVB-N component gases (ammonia, trimethylamine, dimethylamine, propylamine, and triethylamine) and their mixed gases (simulating TVB-N gas), the sensitivity, linear range, accuracy, and stability of TVB-N volatilized from spiked pork and spiked fish samples were evaluated. On this basis, the reliability of Jug@AG hydrogel for dynamic monitoring of the freshness of actual pork and fish samples under room temperature (25 °C) and cold storage (4 °C) conditions was further investigated.

## 2. Materials and Methods

### 2.1. Materials and Reagents

Agarose was purchased from Wuhan Servicebio Technology Co., Ltd. (Wuhan, China). Juglone was purchased from Shanghai Yuanfan Biotechnology Co., Ltd. (Shanghai, China). Anhydrous ethanol, Ammonia (25 wt%), trimethylamine aqueous solution (35 wt%), dimethylamine aqueous solution (2 mol/L), propylamine (99 wt%), triethylamine (99 wt%), acetic acid (99.5 wt%), acetone (99.5 wt%), hexanal (99 wt%), and methanethiol (10% (*w*/*w*)) were all purchased from Shanghai Macklin Biochemical Co., Ltd. (Shanghai, China). Distilled water was obtained from Nanjing Wobio Biotechnology Co., Ltd. (Nanjing, China). Qualitative filter paper with medium filtration speed and polyvinylidene fluoride (PVDF) membrane with the pore size of 0.22 μm and the thickness of 0.1 mm were obtained from Membrane Solutions Biotechnology Co., Ltd. (Nantong, China).

### 2.2. Preparation of Working Solutions

The feasibility of colorimetric sensing of TVB-N gases using the Jug@AG hydrogel in the laboratory-made gas sensing chamber (described in Section 2.5.1) was investigated. The five typical TVB-N component gases (ammonia, trimethylamine, dimethylamine, propylamine, and triethylamine) to be detected and the mixed gases (simulated TVB-N gas) were generated by volatilizing the prepared target solutions in the sensing chamber. Therefore, corresponding working solutions were prepared. The certain volumes of ammonia, trimethylamine, triethylamine, dimethylamine, and propylamine were accurately measured and diluted with distilled water to prepare 50 mg/mL stock solutions, respectively. Then, these stock solutions were serially diluted with distilled water to obtain working solutions of each target at concentrations of 30 mg/mL, 20 mg/mL, 15 mg/mL, 10 mg/mL, 5 mg/mL, 3.75 mg/mL, 2.5 mg/mL, 1.25 mg/mL, 0.5 mg/mL, 0.25 mg/mL, and 0.05 mg/mL, respectively. TVB-N is not a single compound, but a mixture of multiple volatile nitrogen-containing gases, with ammonia having the highest content, followed by trimethylamine, and then other gases such as triethylamine and dimethylamine [1]. Based on the proportion of each component in TVB-N, working solutions of ammonia, trimethylamine, triethylamine, dimethylamine, and propylamine at specific concentrations were mixed in a volume ratio of 65:20:5:5:5 to obtain mixed solutions with corresponding concentrations. The mixed solutions were volatilized in the laboratory-made gas sensing chamber described in Section 2.5.1, and the resulting mixed gas was generated to simulate TVB-N.

### 2.3. Samples Collection and Processing

Five-hundred g each of pork and basa fish were purchased from a local supermarket in Nanjing. The baseline TVB-N contents were measured on the day of purchase via the automated Kjeldahl method, referring to the Chinese National Food Safety Standard “Determination of Total Volatile Basic Nitrogen in Foods” (GB 5009.228-2016) [30]. Then, 200 g each of pork and basa fish were thoroughly homogenized to obtain minced samples, which were further processed into spiked samples, as described in Section 2.5.3, to evaluate the detection efficiency of volatile TVB-N using Jug@AG hydrogel. The remaining 300 g of pork and basa fish were cut into cubed samples with sterile knives for reliability studies on dynamic freshness monitoring of actual samples (details were described in Section 2.6).

### 2.4. Preparation and Characterization of Jug@AG Hydrogel

A specific amount of agarose was accurately weighed and added to distilled water, then heated at 80 °C until completely dissolved to obtain a 2% (*w*/*v*) agarose solution. Juglone was also accurately weighed and completely dissolved in anhydrous ethanol to obtain a 0.12% (*w*/*v*) juglone solution.

The agarose and juglone solutions were thoroughly mixed in a volume ratio of 9:1, and a certain volume of the mixed solution was poured into the petri dish. After cooling and solidifying at room temperature, the Jug@AG hydrogel with a specific thickness was formed. The Jug@AG hydrogel was then cut into circular discs with a diameter of 1 cm using a hole puncher and stored in brown bottles at 4 °C. The concentration of agarose and juglone solutions and the thickness of Jug@AG hydrogel were optimized. The tested agarose solution concentrations were 1%, 1.5%, 2%, 2.5%, and 3%, while the juglone solution concentrations were 0.04%, 0.06%, 0.08%, 0.1%, 0.12%, 0.14%, and 0.16%. The tested hydrogel thicknesses were 1 mm, 1.5 mm, 2 mm, 2.5 mm, and 3 mm.

To comprehensively evaluate the potential advantages of Jug@AG hydrogels for TVB-N colorimetric sensing, juglone was immobilized onto PVDF membranes and filter papers to fabricate PVDF membrane-based (Jug@PVDF) and filter paper-based (Jug@FP) solid-phase sensors, respectively. The specific methods were shown as follows: 20 μL of juglone solution (5 mg/mL) was dropped onto 1-cm-diameter filter paper and PVDF discs to achieve the same juglone loading as that in Jug@AG hydrogel. After evaporating the solvent at room temperature, Jug@PVDF and Jug@FP were obtained. Ammonia was used as the model target, and the colorimetric sensing performances of Jug@AG, Jug@PVDF, and Jug@FP were compared following the procedures described in Section 2.5.2.

The morphologies of Jug@AG, Jug@PVDF, and Jug@FP were observed using a scanning electron microscope (SEM) (S-4800, Hitachi, Tokyo, Japan). The porosities were determined by mercury porosimeter (AutoPore V9620, Micromeritics, Norcross, GA, USA). The viscoelasticity of Jug@AG was analyzed using a rheometer (MCR92, Anton Paar, Graz, Austria).

### 2.5. Evaluation of Jug@AG Hydrogel as a Colorimetric Sensor for TVB-N

#### 2.5.1. Laboratory-Made Gas Sensing Chamber

**Figure 1:**
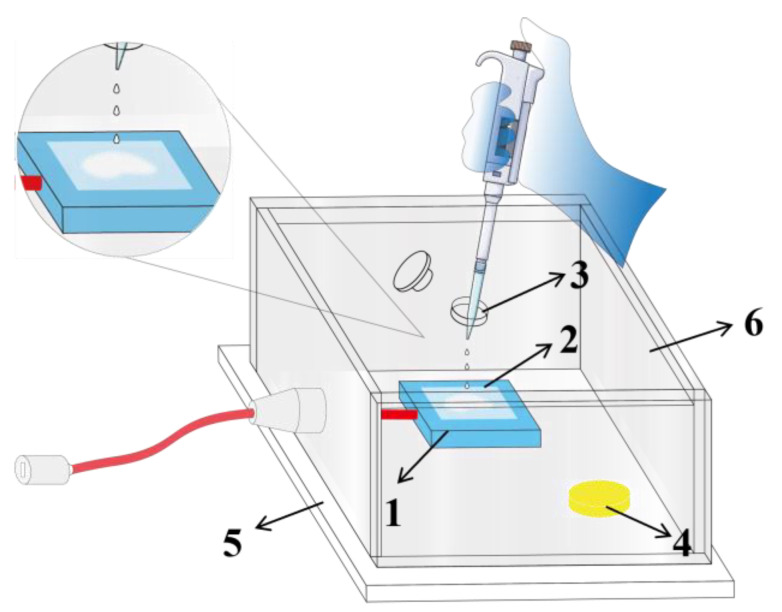
Schematic diagram of the sensing device based on Jug@AG hydrogel. 1 heating plate, 2 sample tray, 3 sample loading port, 4 Jug@AG hydrogel, 5 base, 6 openable upper chamber.

As shown in Figure 1, the laboratory-made gas sensing chamber consists of a base and an openable upper chamber, both of which are made of transparent acrylic material with a thickness of 5 mm. The internal length, width, and height are 10 cm, 10 cm, and 5 cm, respectively, with an inner chamber volume of 500 cm^3^. An aluminum plate with dimensions of 3.5 cm (length) × 2 cm (width) × 0.5 cm (thickness) is positioned within the chamber and can reach a constant temperature of 170 °C within 40 s after being powered on. A heat-conducting quartz glass sheet with dimensions of 2 cm × 2 cm × 0.5 mm is placed on the aluminum heating plate as the sample tray. A circular hole with a diameter of 2 cm aligned with the sample tray serves as the sample loading port, where the port plug is sealed with a rubber sealing ring to ensure air-tightness within the chamber.

#### 2.5.2. Investigation of the Feasibility of the Jug@AG Hydrogel as a Colorimetric Sensor for TVB-N

The feasibility of the Jug@AG hydrogel as a TVB-N colorimetric sensor was evaluated by investigating its ability to achieve rapid and recognizable color changes with the naked eye in a TVB-N gas atmosphere. Based on the investigation of its sensing performance toward five representative TVB-N component gases (ammonia, trimethylamine, triethylamine, dimethylamine, and propylamine), the sensing efficiency for simulated TVB-N was further evaluated.

The chamber was opened from the side not connected to the heating plate, and the Jug@AG hydrogel was placed at least 3 cm away from the aluminum heating plate. A volume of 100 μL of each target working solution or mixed working solution (prepared as described in Section 2.2) was deposited onto the sample tray through the sample loading port, followed by sealing the chamber and powering on the aluminum plate. The atmospheres of ammonia, trimethylamine, triethylamine, dimethylamine, propylamine, or a mixture were formed within the chamber after evaporating the solvent for 2 min. The concentrations (mg/dm^3^) of these targets were calculated based on the solution concentrations and the internal volume of the chamber. After the solution was completely evaporated (i.e., 2 min after the aluminum plate was powered on), the color change of the Jug@AG hydrogel was observed. A smartphone was horizontally placed above the sensing chamber to capture images of the Jug@AG hydrogel. The RGB color analysis was performed using Image J v1.8.0.112 software, and the color difference value (∆C) was calculated with Equation (1). The time required to reach a stable ∆C was recorded. A nonlinear curve fitting method was employed to establish the response relationship between the ∆C value and the target gas concentration. The goodness of fit was evaluated through R^2^ and residual analysis to ensure no systematic bias.

(1) $$\Delta C=\sqrt{2{\left(R_{1}-R_{2}\right)}^{2}+4(G_{1}-G_{2}{)}^{2}+3{\left(B_{1}-B_{2}\right)}^{2}+\frac{{\left(\frac{R_{1}+R_{2}}{2}\right)}^{2}((R_{1}-R_{2})-(B_{1}-B_{2}))}{256}}$$

where R_1_, G_1_, B_1_ and R_2_, G_2_, B_2_ represented the RGB values of the Jug@AG hydrogel before and after colorimetric sensing, respectively.

#### 2.5.3. Evaluation of the Efficiency of Jug@AG Hydrogel for Detecting TVB-N Volatilized from Samples

Then, 100 μL of the mixed working solution with corresponding concentrations was added to 10 g of pork or fish mince, which was homogenized using a homogenizer for 30 s. The total added amounts of the 5 main components of TVB-N (the following is named as simulated TVB-N addition amounts) in the spiked pork and fish samples were 5 mg/100 g, 10 mg/100 g, 15 mg/100 g, 20 mg/100 g, 30 mg/100 g, and 50 mg/100 g, respectively. After placing the Jug@AG hydrogel in the sensing chamber, as described in Section 2.5.2, the prepared spiked pork or fish samples were placed on the sample tray through the sample loading port, and the sensing chamber was immediately sealed. The aluminum plate was powered on for 2 min to volatilize each target to form a simulated TVB-N atmosphere. Then, a photo of the Jug@AG hydrogel was taken using a smartphone, and ∆C was calculated by Equation (1). A working curve was constructed between ∆C and the simulated TVB-N addition amounts mentioned above. The lowest point of the working curve was also used as the lowest observable concentration to evaluate sensitivity.

To evaluate accuracy and precision, spiked pork and fish samples were prepared at low, medium and high levels (5 mg/100 g, 20 mg/100 g, and 50 mg/100 g). The experiment was conducted according to Section 2.5.2. The obtained ∆C were substituted into the working curve to calculate the measured amount of TVB-N, and then the recovery rate at each addition level was calculated using Equation (2). The measured amount of TVB-N in spiked samples was detected within one day, and the relative standard deviation (RSD) was calculated to evaluate the intraday precision (n = 3). The interday precision was evaluated by calculating the RSD of TVB-N in spiked samples over three consecutive days.

(2) $$R(\%)=\frac{\bar{C}}{C_{0}}\times 100\%$$

where (mg/100 g) was the measured amount of TVB-N, and (mg/100 g) was the corresponding TVB-N addition level.

The Jug@AG hydrogel was placed in a brown bottle and stored in refrigerator at 4 °C to investigate the storage stability. According to the procedures described in Section 2.5.2, the spiked pork and fish samples with a simulated TVB-N amount of 50 mg/100 g were daily detected within 7 days. The ΔC values of the Jug@AG hydrogel were calculated using Equation (1)., and statistical evaluation was performed through one-way analysis of variance (ANOVA) using IBM SPSS statistics 27 software. The means were considered significantly different at *p* < 0.05.

To evaluate the specificity of the Jug@AG hydrogel for detecting TVB-N, other primary volatile compounds released during meat spoilage (methanethiol, hexanal, acetone, acetic acid, and ethanol) at a concentration of 10 mg/dm^3^ were detected following the procedure described in Section 2.5.2. The ΔC values of the Jug@AG hydrogel were calculated using Equation (1).

### 2.6. Reliability Evaluation of Jug@AG Hydrogel for Dynamic Monitoring of Actual Sample Freshness

**Figure 2:**
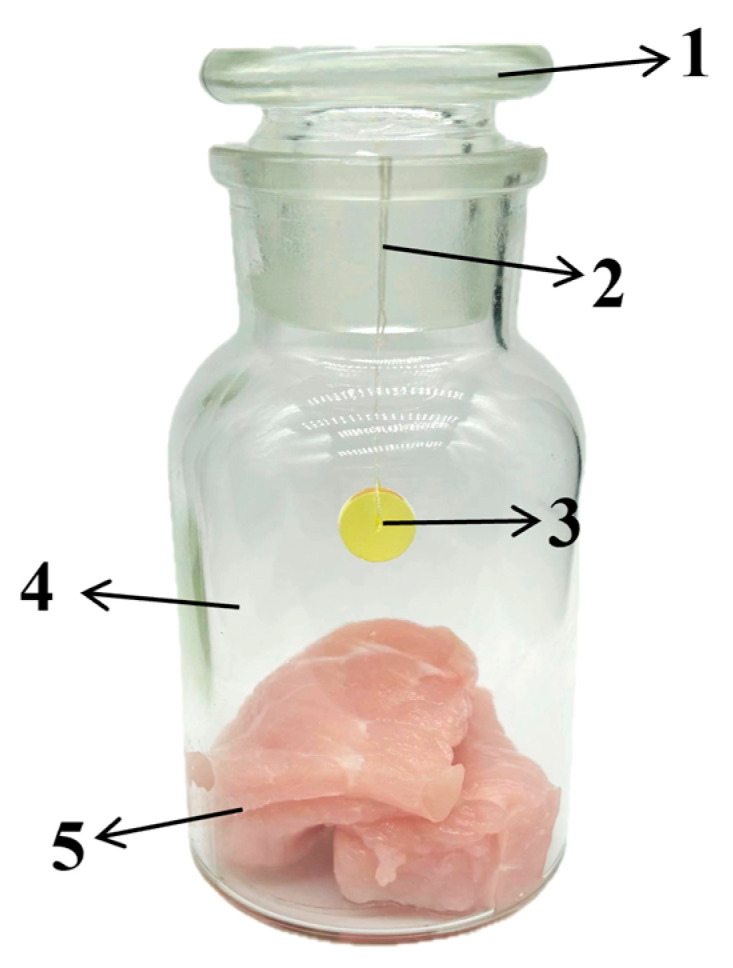
Schematic diagram of Jug@AG hydrogel for actual sample detection. 1 bottle stopper, 2 sterilized nylon thread, 3 Jug@AG hydrogel, 4 glass wide-mouth bottle, 5 actual sample.

As shown in Figure 2, 60 ± 1.5 g of pork or fish samples are weighed and transferred to 125 mL clean glass wide-mouth bottles, respectively. A sterilized nylon thread is used to vertically pass through the center of the Jug@AG hydrogel, which is suspended 2 cm above the meat samples. Medical-grade Vaseline was uniformly spread on the inside of the glass bottle stopper, which was screwed tightly to enhance the air tightness. Then, the wide-mouth bottles were stored at room temperature (25 °C) and cold storage (4 °C) conditions for 72 h and 96 h, respectively. During this period, the images of the Jug@AG hydrogel were captured using a smartphone every 6 h, and the ΔC values of the hydrogel were calculated using Equation (1). According to the Chinese National Food Safety Standards of “Determination of Total Volatile Basic Nitrogen in Foods” (GB 5009.228-2016) [30] and “Microbiological Examination of Food—Detection of aerobic bacterial count” (GB 4789.2-2016) [31], the automatic Kjeldahl nitrogen determination method and the plate counting method were employed to detect TVB-N and TVC in pork and fish during the experimental monitoring period, respectively. The reliability of the Jug@AG hydrogel for dynamic monitoring of actual sample freshness was assessed based on the consistency between trend variations in the hydrogel’s ΔC values and the measured TVB-N and TVC levels in pork and fish.

## 3. Results and Discussion

### 3.1. Comparison of Jug@AG Hydrogel, Jug@PVDF, and Jug@FP

**Figure 3:**
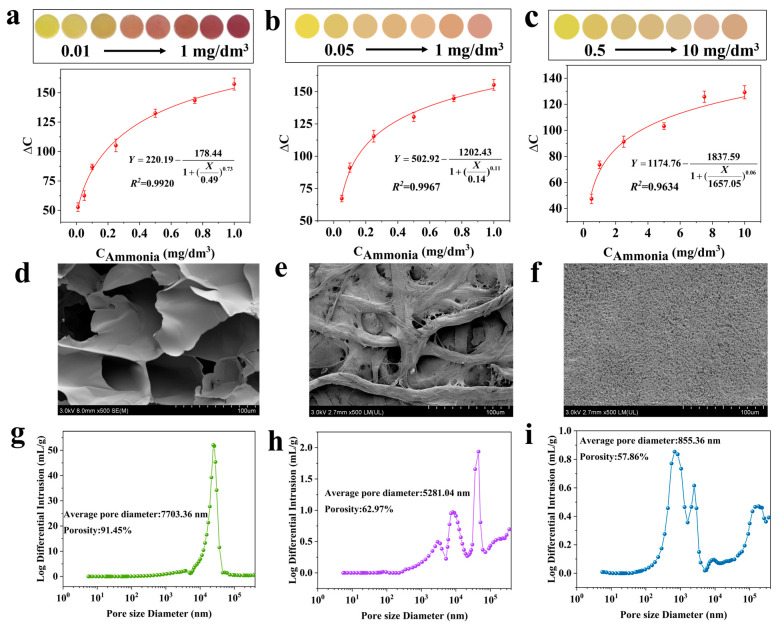
Color change images and fitting curves of ΔC values (Y) against gas concentration (X) for ammonia sensing using (**a**) Jug@AG hydrogel, (**b**) Jug@FP, and (**c**) Jug@PVDF; (**d**) SEM images of Jug@AG hydrogel, (**e**) Jug@FP, and (**f**) Jug@PVDF; (**g**) pore size distribution plots of Jug@AG hydrogel, (**h**) Jug@FP, and (**i**) Jug@PVDF.

As shown in Figure 3a–c, the initial colors of Jug@AG hydrogel, Jug@PVDF, and Jug@FP exhibit a consistent yellow color with the same addition amount of juglone. The ∆C values of the Jug@AG hydrogel vary from 52.77 to 157.33 across ammonia concentrations ranging from 0.01 mg/dm^3^ to 1 mg/dm^3^, accompanied by a wide color variation of yellow-brownish-purplish red (Figure 3a). When ammonia concentrations range from 0.05 mg/dm^3^ to 1 mg/dm^3^, the variation ranges of the ∆C value of Jug@FP are 67.29–155.26, showing only a narrow chromaticity change of yellow-light orange-pink (Figure 3b). It is shown in Figure 3c that Jug@PVDF requires a high concentration range of 0.5–10 mg/dm^3^ to achieve ΔC value variations of 47.42–129.29, exhibiting a narrow chromaticity change similar to Jug@FP. In addition, as shown in Figure S1a–c, the residual plots show no systematic deviation, and the correlation coefficients R^2^ between the gas concentrations and ΔC values are all greater than 0.95, indicating a good curve-fitting effect. The results demonstrate that juglone exhibits the ability to serve as a colorimetric probe for TVB-N. When juglone is immobilized on different carriers to prepare solid-phase colorimetric sensors, only Jug@AG hydrogel exhibits a broad range of chromaticity change, which is more conducive to visual detection of color changes. Additionally, the lowest observable concentration of ammonia for the Jug@AG hydrogel is 0.01 mg/dm^3^, which is significantly lower than 0.05 mg/dm^3^ of Jug@FP and 0.5 mg/dm^3^ of Jug@PVDF, indicating that the Jug@AG hydrogel exhibits higher sensitivity for ammonia detection. The above results suggest that the hydrogel is the superior carrier for probe immobilization.

The advantages of the Jug@AG hydrogel in colorimetric sensing may be attributed to its microstructure. The SEM images show that the Jug@AG hydrogel exhibits an irregular sheet-like morphology with lamellae stacked on each other and a rough surface (Figure 3d). The Jug@AG hydrogel generally reveals a complex three-dimensional spatial structure with an average pore diameter of 7703.36 nm and a porosity of 91.45% (Figure 3g). Jug@FP displays a distinct fibrous structure, where the filter paper fibers crisscross to form an irregular network morphology (Figure 3e), with an average pore size of 5281.04 nm and a porosity of 62.97% (Figure 3h). As for Jug@PVDF, it presents a dense microstructure with relatively small and uniformly distributed surface pores (Figure 3f), exhibiting an average pore size of 855.36 nm and a porosity of 57.86% (Figure 3i). It is obvious that the average pore sizes and porosities of Jug@FP and Jug@PVDF are significantly lower than those of the Jug@AG hydrogel. The large pore size and porous structure of the Jug@AG hydrogel not only reduce the steric effect but also increase its contact area with target gas molecules, which is beneficial for achieving a colorimetric response. Additionally, the dynamic rheological result of Jug@AG hydrogel is shown in Figure S2. The storage modulus (G’) is always higher than the loss modulus (G”), which suggests that it has the characteristics of a viscoelastic solid. Moreover, G’ remains stable within the test frequency range. The excellent mechanical stability of its network structure is conducive to maintaining the morphology of Jug@AG hydrogel during application.

### 3.2. Optimization of the Preparation Conditions for Jug@AG Hydrogel

**Figure 4:**
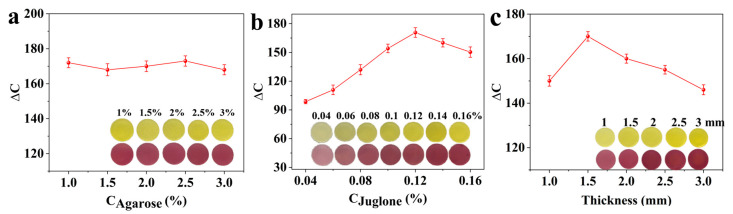
Color change images and ΔC values of Jug@AG hydrogels prepared with (**a**) different agarose solution concentrations (juglone solution concentration: 0.12%, hydrogel thickness: 1.5 mm), (**b**) different juglone solution concentrations (agarose solution concentration: 2%, hydrogel thickness: 1.5 mm), and (**c**) different hydrogel thicknesses (agarose solution concentration: 2%, juglone solution concentration: 0.12%) for ammonia sensing at the concentration of 1 mg/dm^3^.

As shown in Figure 4a, the Jug@AG hydrogel exhibits a naked-eye-visible color change from yellow to purplish red in ammonia atmosphere. With increasing agarose solution concentration, no significant differences in ΔC values are observed (*p* > 0.05), indicating that agarose solutions of different concentrations have little effect on the sensing performance. However, the gelation capability of the Jug@AG hydrogel becomes insufficient for proper molding when the agarose concentration falls below 1.5% (Figure S3). Therefore, a 2% agarose solution is selected to prepare Jug@AG hydrogel. Figure 4b demonstrates that the ∆C value of Jug@AG hydrogel increases with the increase of juglone solution concentration in the range of 0.04–0.12%. However, when the juglone concentration is further elevated from 0.12% to 0.16%, a statistically significant reduction in ∆C value is observed (*p* < 0.05). Therefore, the optimal juglone concentration is determined to be 0.12%.

The ∆C value of the Jug@AG hydrogel initially increases and then decreases, reaching the maximum (∆C = 170.36 ± 2.13) at a thickness of 1.5 mm (Figure 4c). This is likely attributed to the limited contact between ammonia and juglone when the hydrogel thickness exceeds a certain threshold. The response speed of Jug@AG hydrogels with different thicknesses was studied. As shown in Figure S4, the thicker hydrogels exhibit longer response times. Specifically, at a thickness of 1.5 mm, the maximum ∆C value of the Jug@AG hydrogel is reached within 3 min. Therefore, the thickness of Jug@AG hydrogel is selected as 1.5 mm to obtain the optimal response speed and sensitivity.

### 3.3. Evaluation of Jug@AG Hydrogel as the Colorimetric Sensor for TVB-N

#### 3.3.1. Feasibility of Jug@AG Hydrogel as the Colorimetric Sensor for TVB-N

**Figure 5:**
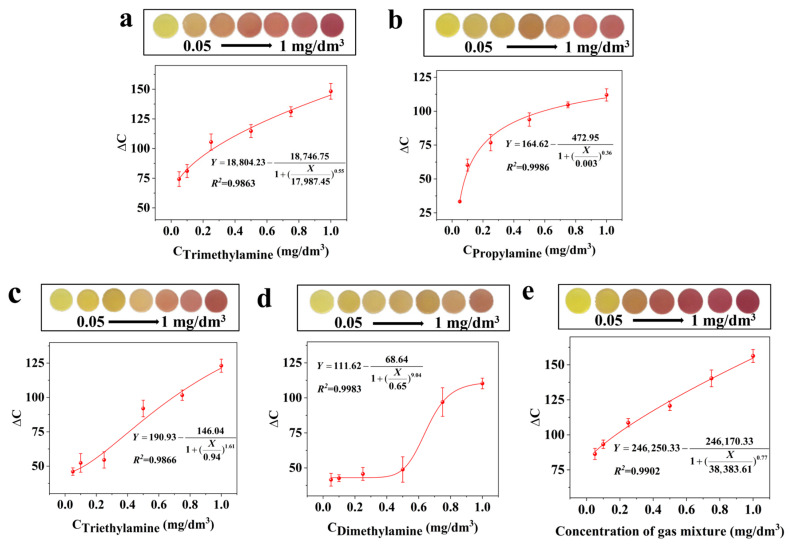
Color change images of the Jug@AG hydrogel during sensing of (**a**) trimethylamine, (**b**) propylamine, (**c**) triethylamine, (**d**) dimethylamine, and (**e**) mixed gases, along with the curve fitting relationships between ΔC values (Y) and gas concentrations (X).

The Jug@AG hydrogel exhibits visible to the naked eye color changes when exposed to five typical TVB-N component gases: ammonia, trimethylamine, dimethylamine, propylamine, and triethylamine (Figure 3a and Figure 5a–d). The overall color transition is from yellow to brownish and then to purplish red. Tests were further conducted on the 5 component gases at different concentrations (0.05 mg/dm^3^, 0.1 mg/dm^3^, 0.25 mg/dm^3^, 0.5 mg/dm^3^, 0.75 mg/dm^3^, 1 mg/dm^3^). It is found that the ΔC values of the Jug@AG hydrogel show good curve relationships with the concentrations of each gas (R^2^ > 0.95), and residual analysis reveals no systematic bias (Figure S5a–d). The lowest observable concentration for ammonia is 0.01 mg/dm^3^, and for other gases is 0.05 mg/dm^3^. As shown in Figures S6–S10, the time required for the ∆C value of Jug@AG hydrogel to be stable is 8 min at a gas concentration of 0.05 mg/dm^3^, and the time significantly shortens to 3 min with the concentration increasing to 1 mg/dm^3^. The sensing time decreases with increasing gas concentration. Among the five typical TVB-N component gases at the same concentration, the Jug@AG hydrogel exhibits the most significant color change upon exposure to ammonia with the largest ΔC variation range (52.77–157.33). Trimethylamine follows with the second-largest range (74.25–148.24), while triethylamine (46.04–123.08), propylamine (33.37–112.01), and dimethylamine (41.54–110.26) show progressively smaller ΔC variations in sequence. The results demonstrate that the Jug@AG hydrogel can effectively detect various representative components of TVB-N, especially with high sensitivity to ammonia (the main component of TVB-N).

The results of the Jug@AG hydrogel exposed to simulated TVB-N gas also exhibit visible to the naked eye color changes from yellow to brownish and then to purplish red (Figure 5e). The curve fitting of the ΔC values of the Jug@AG hydrogel as a function of the simulated TVB-N gas concentration (0.05–1 mg/dm^3^) yields an R^2^ of 0.9902, and the residual plot (Figure S5e) shows no systematic bias, indicating a good fit. The lowest observable concentration of the Jug@AG hydrogel for simulated TVB-N gas is as low as 0.05 mg/dm^3^. Meanwhile, the time required for the ∆C value to reach stability is also shortened from 8 min at a low concentration (0.05 mg/dm^3^) to 3 min at a high concentration (1 mg/dm^3^) (Figure S11). The results indicate that the Jug@AG hydrogel exhibits comparable colorimetric sensing sensitivity and speed under simulated TVB-N gas atmospheres at equivalent concentrations to the five aforementioned TVB-N component gases. The Jug@AG hydrogel demonstrates promising potential as a TVB-N colorimetric sensor.

#### 3.3.2. Evaluation of the Efficiency of Jug@AG Hydrogel for Detecting Volatile TVB-N from Samples

**Figure 6:**
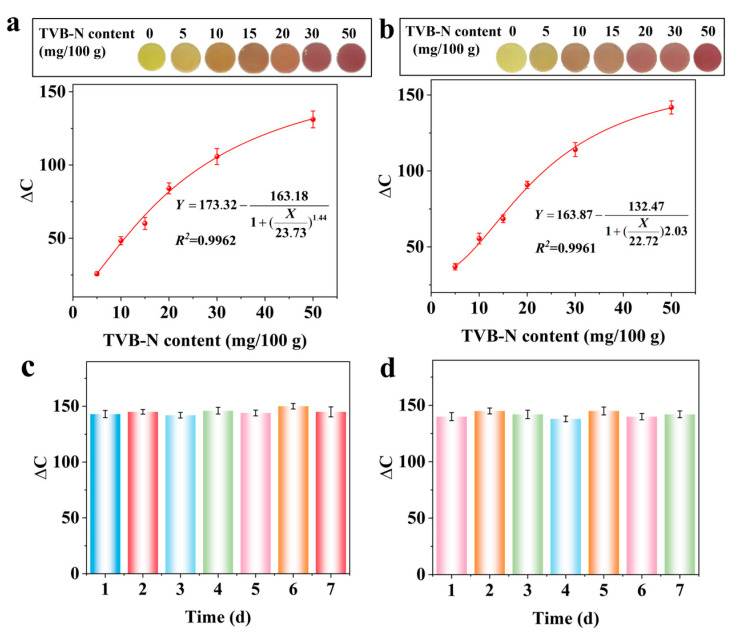
Color change images and curve fitting relationships between ΔC values and addition amounts (5–50 mg/100 g) for (**a**) spiked pork and (**b**) fish; ΔC values for detecting (**c**) spiked pork and (**d**) fish with a 50 mg/100 g addition amount after different storage times.

To verify the practical application potential of the Jug@AG hydrogel as a TVB-N colorimetric sensor, methodological studies were conducted using spiked pork and fish samples. The background values of TVB-N in pork and fish are measured to be 3.43 ± 0.13 mg/100 g and 3.27 ± 0.09 mg/100 g, respectively, consistent with the standards of fresh meat [9]. The Jug@AG hydrogel exhibits naked-eye visible color changes from yellow to brownish to purplish red when exposed to TVB-N gases volatilized from spiked pork and fish samples (Figure 6a,b). The ΔC values of the Jug@AG hydrogel shows a strong correlation in curve fitting with the simulated TVB-N amounts (5–50 mg/100 g) in both spiked pork and fish samples, with R^2^ values of 0.9962 and 0.9961, respectively. The high reliability of the curve fitting is verified by the distribution characteristics of the residual plot without systematic bias (Figure S12). Combined with the TVB-N background values, the lowest observable amounts for spiked pork and fish are calculated to be 8.43 mg/100 g and 8.27 mg/100 g at the minimum addition amount of TVB-N (5 mg/100 g), which are lower than the early spoilage limit of 10 mg/100 g for animal-derived food. Meanwhile, the ΔC values increases from 0 to 29.82 ± 1.05 for spiked pork and from 0 to 36.97 ± 1.13 for spiked fish. The Jug@AG hydrogel exhibits the naked-eye visible color changes from yellow to light grayish yellow, demonstrating its potential for visual detection of early spoilage in pork and fish.

The average recoveries for spiked pork and fish samples at three levels (5 mg/100 g, 20 mg/100 g, and 50 mg/100 g) are 87.72–92.53% and 91.35–96.17%, respectively (Table 1). The intraday RSDs are measured to be 6.18–8.02% and 5.87–7.22%, while the interday RSDs range from 8.16–9.36% and 7.37–9.05%, demonstrating good accuracy and precision of the method. After the Jug@AG hydrogel is stored at 4 °C for 7 days, its ΔC values for detection of spiked pork samples remain stable within 142–150 (Figure 6c) and 140–145 for spiked fish samples (Figure 6d). No significant differences are observed in ΔC values across different storage times (*p* > 0.05), indicating that the Jug@AG hydrogel maintains stable sensing efficiency after 7 days of storage.

**Figure 7:**
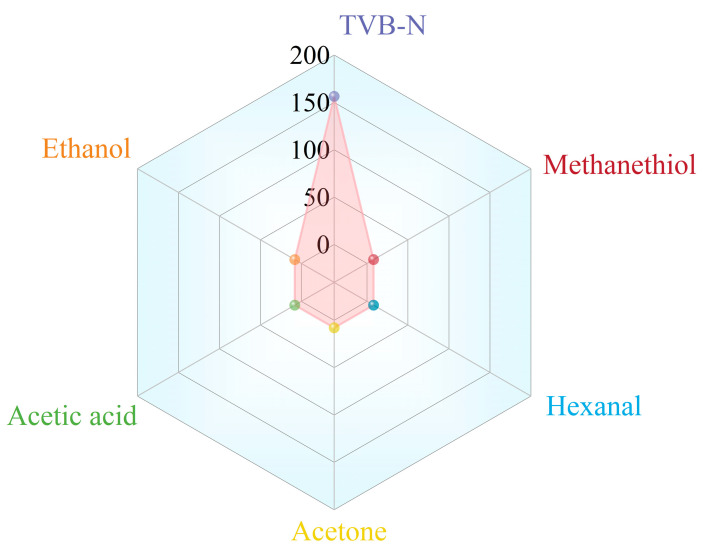
The colorimetric monitoring of gases possibly generated during the meat spoilage by Jug@AG hydrogel (simulated TVB-N at a concentration of 1 mg/dm^3^, as mentioned before, and other volatile compounds at 10 mg/dm^3^).

As shown in Figure 7, the Jug@AG hydrogel exhibits a significant colorimetric response to TVB-N, a key indicator of meat spoilage, while showing no response to common spoilage volatile compounds such as methanethiol, hexanal, acetic acid, ethanol, and acetone. This indicates that the hydrogel can effectively distinguish the target gas from interfering components and is suitable for the specific detection of TVB-N content.

### 3.4. Reliability Assessment of the Jug@AG Hydrogel for Dynamic Monitoring of Actual Sample Freshness

**Figure 8:**
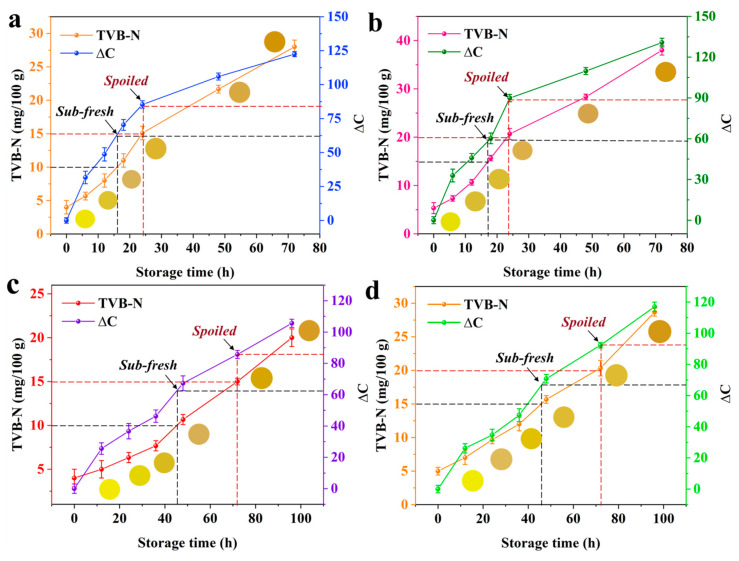
Growth trends of TVB-N and ΔC values of the Jug@AG hydrogel for pork and fish samples at different storage temperatures: (**a**) pork and (**b**) fish stored at room temperature (25 °C), and (**c**) pork and (**d**) fish stored at cold storage (4 °C).

As shown in Figure 8a,b, the TVB-N contents of pork and fish are 5.67 ± 0.88 mg/100 g and 7.33 ± 0.55 mg/100 g with TVC contents of 4.35 ± 0.22 lg (CFU/g) and 4.6 ± 0.3 lg (CFU/g) after storage at room temperature (25 °C) for 6 h, which indicate that both samples remain fresh. The ΔC values of the Jug@AG hydrogel are 30.64 ± 0.65 and 33.52 ± 0.29, corresponding to the yellow of the original hydrogel. The pork and fish exhibit TVB-N contents of 8.23 ± 0.46 mg/100 g and 11.15 ± 0.58 mg/100 g alongside TVC contents of 5.13 ± 0.15 lg (CFU/g) and 5.8 ± 0.23 lg (CFU/g) when stored for 12 h. Both samples remain fresh, while the yellow color of the Jug@AG hydrogel deepens further, with ∆C values reaching 44.65 ± 0.55 and 48.3 ± 0.25, respectively. After continuous storage for 18 h, the TVB-N contents of pork and fish increase to 11.0 ± 0.63 mg/100 g and 15.67 ± 0.55 mg/100 g, and the TVC values are 6.04 ± 0.21 lg (CFU/g) and 6.5 ± 0.3 lg (CFU/g), indicating the early spoilage of pork and fish. At this stage, the ΔC values of the Jug@AG hydrogel reach 60.28 ± 2.73 and 70.37 ± 2.95 with its color changing from yellow to grayish-yellow. For 24 h storage, the TVB-N value of pork increases to 15.1 ± 0.18 mg/100 g, and that of fish increases to 20.67 ± 0.58 mg/100 g (Figure S13a,b). The TVC values of pork and fish reach 7.12 ± 0.12 lg (CFU/g) and 7.65 ± 0.30 lg (CFU/g), respectively. Both values exceed the microbial safety limit of 7 lg (CFU/g), confirming complete spoilage. Meanwhile, the ΔC values of the Jug@AG hydrogel increase to 85.37 ± 1.55 and 90.93 ± 2.24, with its color transitioning visibly from grayish-yellow to brownish. The ΔC value variation trend of the Jug@AG hydrogel is consistent with the growth trends of TVB-N and TVC, verifying the reliability of this hydrogel in monitoring freshness. Therefore, the freshness of animal-derived food can be dynamically monitored through color transitions of the Jug@AG hydrogel: yellow represents freshness, grayish-yellow represents early spoilage, and brownish represents complete spoilage.

The change trends of TVB-N, TVC and Jug@AG hydrogel colors for pork and fish at different times under cold storage (4 °C) are shown in the Figure 8c,d. The pork and fish exhibit TVB-N contents of 6.33 ± 0.56 mg/100 g and 9.67 ± 0.48 mg/100 g, respectively, with TVC contents of 4.88 ± 0.31 lg (CFU/g) and 5.23 ± 0.21 lg (CFU/g) after 24 h of storage. All samples remain fresh following 24-h refrigeration. The ΔC values of the Jug@AG hydrogel are measured as 36.69 ± 0.91 and 40.3 ± 0.23, while the hydrogel maintains its yellow coloration. After 48 h of storage, the TVB-N contents of pork and fish reach 10.6 ± 0.58 mg/100 g and 15.5 ± 0.43 mg/100 g, respectively, accompanied by TVC values of 6.15 ± 0.11 lg (CFU/g) and 6.43 ± 0.2 lg (CFU/g). Both the TVB-N and TVC levels in pork and fish have exceeded the limit for early spoilage. Simultaneously, the ΔC values of the Jug@AG hydrogel rise to 64.31 ± 1.56 and 70.80 ± 2.13, with its color transitioning from initial yellow to grayish-yellow. Compared with pork and fish that exhibit early spoilage at 18 h under room temperature, the longer time is required for early spoilage to occur during refrigeration. After 72 h of 4 °C storage, the TVB-N (15.65 ± 0.46 mg/100 g, 20.33 ± 0.37 mg/100 g) and TVC (7.24 ± 0.53 lg (CFU/g), 7.48 ± 0.62 lg (CFU/g)) of pork and fish reach the limit of complete spoilage. This corresponds to the color change of Jug@AG hydrogel from grayish-yellow to brownish, with ∆C values increasing to 85.80 ± 1.36 and 92.28 ± 1.45 (Figure S13c,d). Under refrigeration, the Jug@AG hydrogel demonstrates a slower ΔC increase rate and delayed chromatic transition, consistent with retarded TVC growth at low temperatures. These results align with the common sense that low temperature inhibits meat spoilage. Notably, only a fraction of TVB-N volatilizes into the enclosed space during the storage. Nevertheless, the Jug@AG hydrogel effectively concentrates trace TVB-N, enabling visual detection through sequential color changes from yellow to grayish-yellow and ultimately brownish. The sensor’s sensitivity allows dynamic monitoring of meat quality throughout freshness, early spoilage, and complete spoilage stages.

### 3.5. Comparison with Existing Hydrogel-Based Colorimetric Sensing Methods for TVB-N Content

A total of 33 articles were recovered from the Web of Science database using keywords of “hydrogel”, “colorimetric”, “food”, and “freshness” over the past three years. Among them, 21 articles describe experimental studies on colorimetric sensing for spoilage monitoring of animal-derived foods [2,15,16,17,18,19,20,21,25,26,32,33,34,35,36,37,38,39,40,41,42]. Therefore, they were included in the literature comparison. In this study, a novel solid-phase colorimetric sensor, Jug@AG hydrogel, was developed to investigate its sensing performance toward five typical TVB-N component gases: ammonia, trimethylamine, dimethylamine, propylamine, and triethylamine, and their mixed gases. Spiked samples were prepared to evaluate the recovery, precision, and sensitivity of the sensor to ensure the reliability of the TVB-N detection results. The Jug@AG hydrogel demonstrates excellent detection performance in practical applications. When TVB-N content in pork and fish reaches the early spoilage limit of 10 mg/100 g and 15 mg/100 g, respectively, the sensor’s color changes from yellow to grayish-yellow visible to the naked eye. As TVB-N content exceeds the spoilage threshold of 15 mg/100 g, the hydrogel further changes to brownish, enabling real-time dynamic monitoring of animal-derived food freshness.

In contrast, existing studies have not emphasized methodological evaluation through simulated samples. They only use typical TVB-N component gases volatilized from target solutions for detection, while neglecting the influence of sample matrices. It is known that solution matrices cannot represent the actual sample matrices. Therefore, the simulated samples were used for methodological evaluation in this study, which better approximate real application scenarios and enhance the accuracy and reliability of detection results. In practical applications, existing studies have successfully achieved naked-eye colorimetric detection of TVB-N content in livestock, poultry meats, and aquatic products, including quantification thresholds of above 15 mg/100 g in beef [19,32], chicken [2,15,16,21,39], and pork [16,18,35,36,41,42], 20 mg/100 g in freshwater fish [17,37] and shrimp [25,26,33,34,36,38,40], and 30 mg/100 g in marine fish [19,20]. However, the sensitivity of these methods remains inadequate for early spoilage monitoring, falling to meet the required limits of 10 mg/100 g for livestock/poultry meats and 15 mg/100 g for freshwater fish/shrimp.

## 4. Conclusions

The Jug@AG hydrogel was prepared to dynamically monitor the freshness of animal-derived food, which was the first study to explore the application potential of the natural dye juglone for TVB-N colorimetric sensing. The results demonstrate that the Jug@AG hydrogel is feasible for colorimetric sensing of five typical TVB-N component gases (ammonia, trimethylamine, dimethylamine, propylamine, and triethylamine) and their mixed gases (simulated TVB-N gas). Moreover, the Jug@AG hydrogel exhibits good sensitivity, accuracy, and stability in colorimetric sensing of TVB-N volatilized from spiked pork and fish samples. The Jug@AG hydrogel shows the naked-eye visible color variation of yellow- grayish yellow-brownish when pork and fish samples deteriorate from freshness to early spoilage and then to spoilage, confirming its potential for visual dynamic monitoring of animal-derived food freshness.

## Figures and Tables

**Table 1:** Recovery and precision of the Jug@AG hydrogel for colorimetric sensing of TVB-N content in spiked samples.

Samples	TVB-N (mg/100 g, *n* = 3)		Recovery (%)	RSD (%)	
	Addition Levels	Measured Amount		Intraday (*n* = 3)	Interday (*n* = 3)
Spiked pork	5	4.56 ± 0.56	91.26	7.35	8.16
	20	17.54 ± 1.10	87.72	6.18	8.56
	50	46.27 ± 2.65	92.53	8.02	9.36
Spiked fish	5	4.65 ± 0.35	93.08	5.87	7.37
	20	18.27 ± 1.5	91.35	6.57	9.05
	50	48.08 ± 2.05	96.17	7.22	8.08

## Data Availability

The original contributions presented in the study are included in the article/Supplementary Materials, further inquiries can be directed to the corresponding author.

## Supplementary Materials

The following supporting information can be downloaded at: https://www.mdpi.com/article/10.3390/foods14142505/s1, Figure S1. Residual analysis plots for ammonia sensing performance of (a) Jug@AG, (b) Jug@FP, and (c) Jug@PVDF. Figure S2. Changes in storage modulus (G’) and loss modulus (G”) of Jug@AG hydrogel with frequency. Figure S3. Images of Jug@AG hydrogels prepared with agarose solution concentrations of (a) 1.5% and (b) 2%. Figure S4. Response times of Jug@AG hydrogels with different thicknesses for sensing 1 mg/dm^3^ ammonia. Figure S5. Residual analysis plots of Jug@AG hydrogel for (a) trimethylamine, (b) propylamine, (c) triethylamine, (d) dimethylamine, and (e) mixed gases sensing. Figure S6. The ΔC values and images of Jug@AG hydrogel at different response times when sensing ammonia with concentrations of 0.01 mg/dm^3^-1 mg/dm^3^. Figure S7. The ΔC values and images of Jug@AG hydrogel at different response times when sensing trimethylamine with concentrations of 0.01 mg/dm^3^-1 mg/dm^3^. Figure S8. The ΔC values and images of Jug@AG hydrogel at different response times when sensing propylamine with concentrations of 0.01 mg/dm^3^-1 mg/dm^3^. Figure S9. The ΔC values and images of Jug@AG hydrogel at different response times when sensing triethylamine with concentrations of 0.01 mg/dm^3^-1 mg/dm^3^. Figure S10. The ΔC values and images of Jug@AG hydrogel at different response times when sensing of dimethylamine with concentrations of 0.01 mg/dm^3^-1 mg/dm^3^. Figure S11. The ΔC values and images of Jug@AG hydrogel at different response times when sensing mixed gases with concentrations of 0.01 mg/dm^3^-1 mg/dm^3^. Figure S12. Residual analysis plots of (a) spiked pork and (b) spiked fish samples with the simulated TVB- N addition amount in the range of 5 mg/100 g-50 mg/100 g for detection. Figure S13. The variation of TVC of the sample with storage time at different temperatures: (a) pork and (b) fish stored at room temperature (25 °C); (c) pork and (d) fish stored under cold storage (4 °C).
