# Supplementary material for: Visual Colorimetric Sensing of the Animal-Derived Food Freshness by Juglone-Loaded Agarose Hydrogel
Source: Foods. 2025 Jul 17;14(14):2505. doi: 10.3390/foods14142505 (PMC12294289; doi:10.3390/foods14142505)
Supplement: Supplementary file 1 [file foods-14-02505-s001.zip › foods-3701721-supplementary.pdf]

Supplementary Materials

Visual Colorimetric Sensing of the Animal-derived Food Freshness by Juglone-loaded Agarose Hydrogel

Lanjing Wang[a], Weiyi Yan[a], Aijun Li[a], Huayin Zhang[a*], Qian Xu[a*]

[a] Key Laboratory of Environmental Medicine Engineering, Ministry of Education, School of Public Health, Southeast University, Nanjing 210009, China

*Corresponding to:

Qian Xu. Tel.: +86 025 83272563; E-mail: q_xu68@163.com, q-xu@seu.edu.cn.

Huayin Zhang.: E-mail: zhhy940102@126.com.

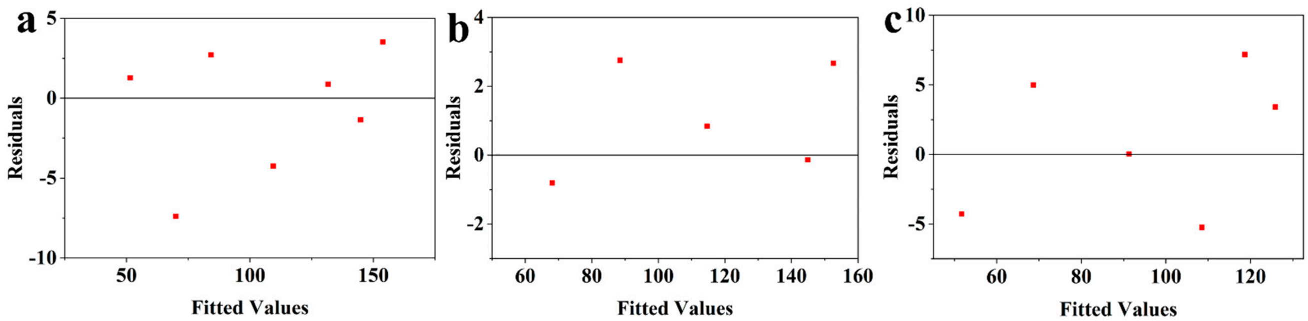

Figure S1. Residual analysis plots for ammonia sensing performance of (a) Jug@AG, (b) Jug@FP, and (c) Jug@PVDF.

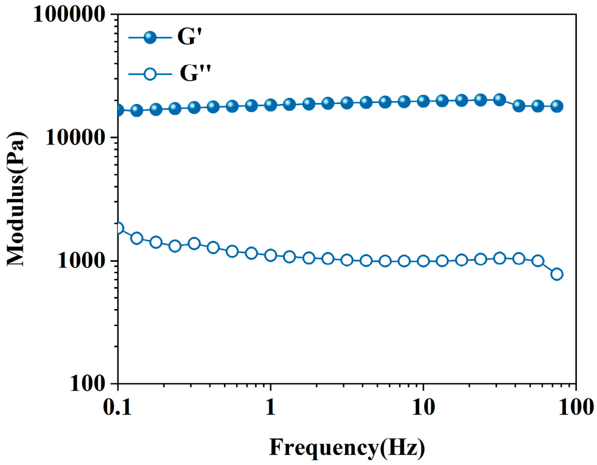

Figure S2. Changes in storage modulus (G') and loss modulus (G'') of Jug@AG hydrogel with frequency.

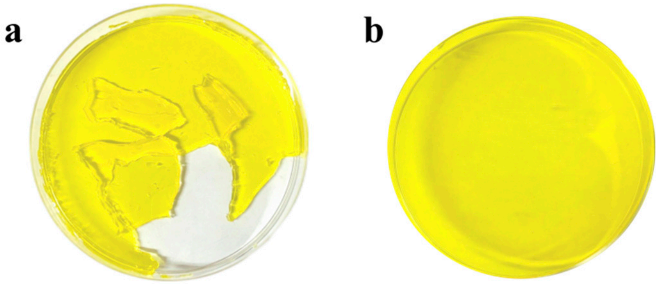

Figure S3. Images of Jug@AG hydrogels prepared with agarose solution concentrations of (a) 1.5% and (b) 2%.

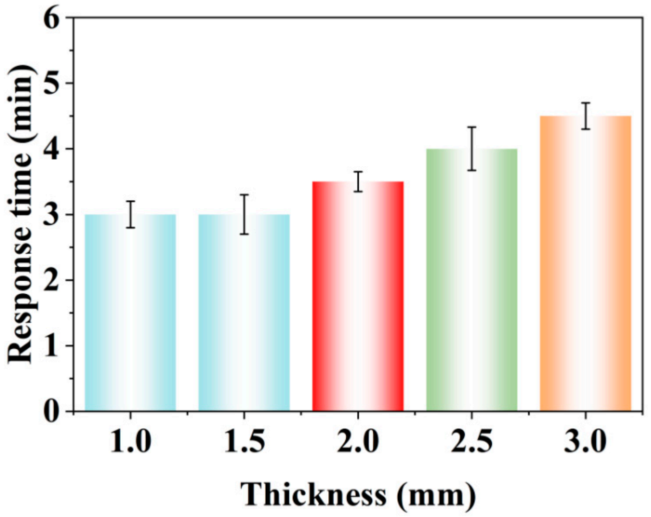

Figure S4. Response times of Jug@AG hydrogels with different thicknesses for sensing 1 mg/dm³ ammonia.

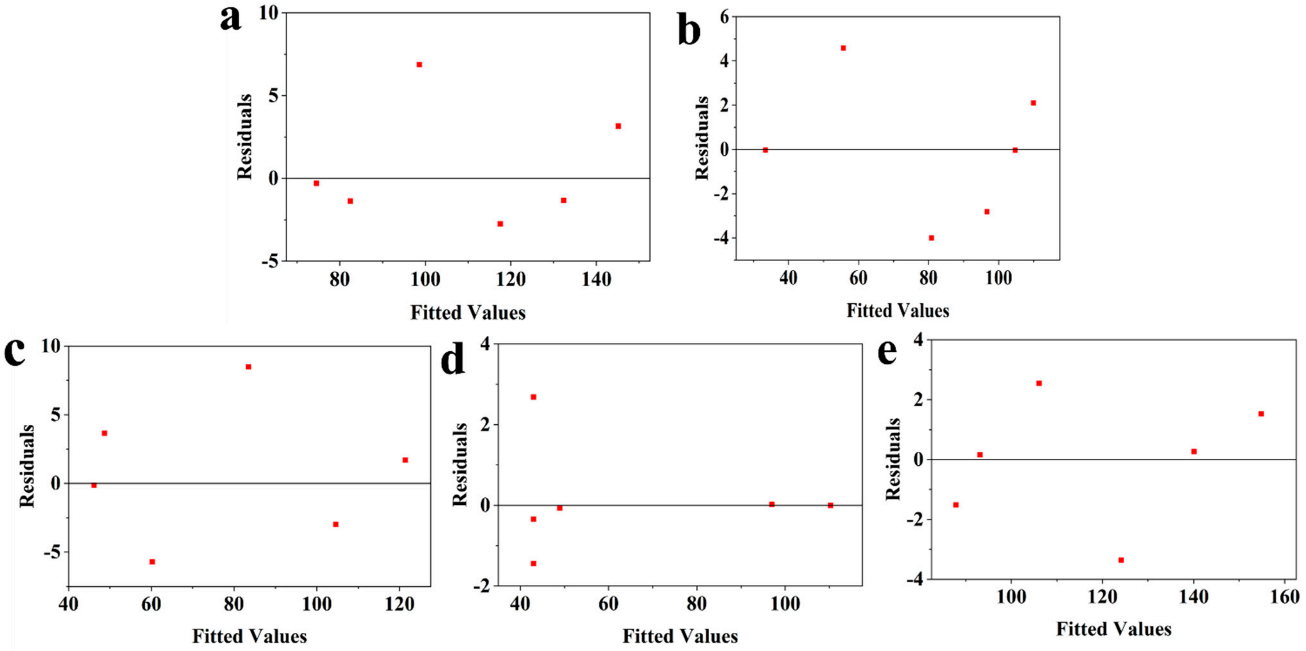

Figure S5. Residual analysis plots of Jug@AG hydrogel for (a) trimethylamine, (b) propylamine, (c) triethylamine, (d) dimethylamine, and (e) mixed gases sensing.

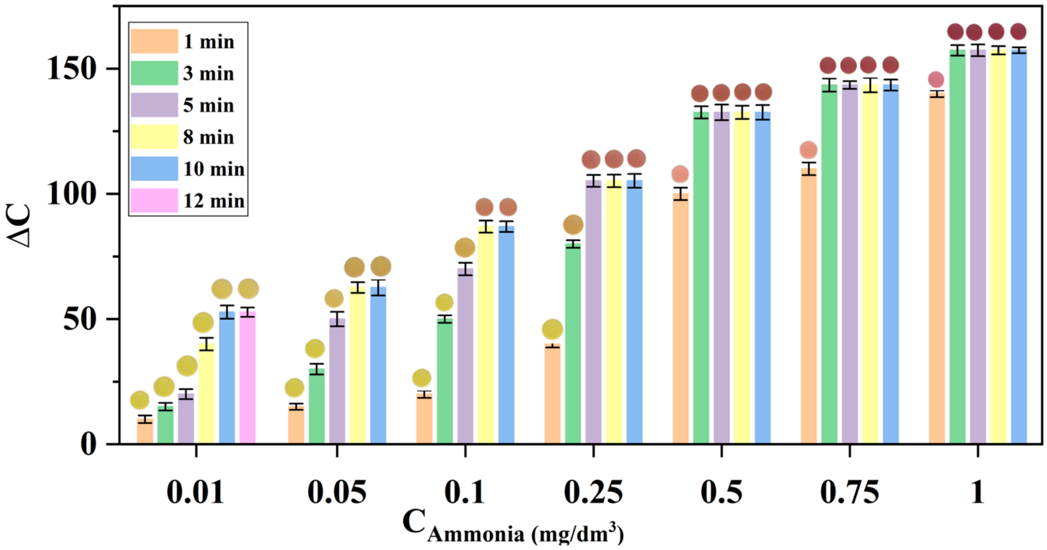

Figure S6. The ΔC values and images of Jug@AG hydrogel at different response times when sensing ammonia with concentrations of 0.01 mg/dm^3-1 mg/dm^3.

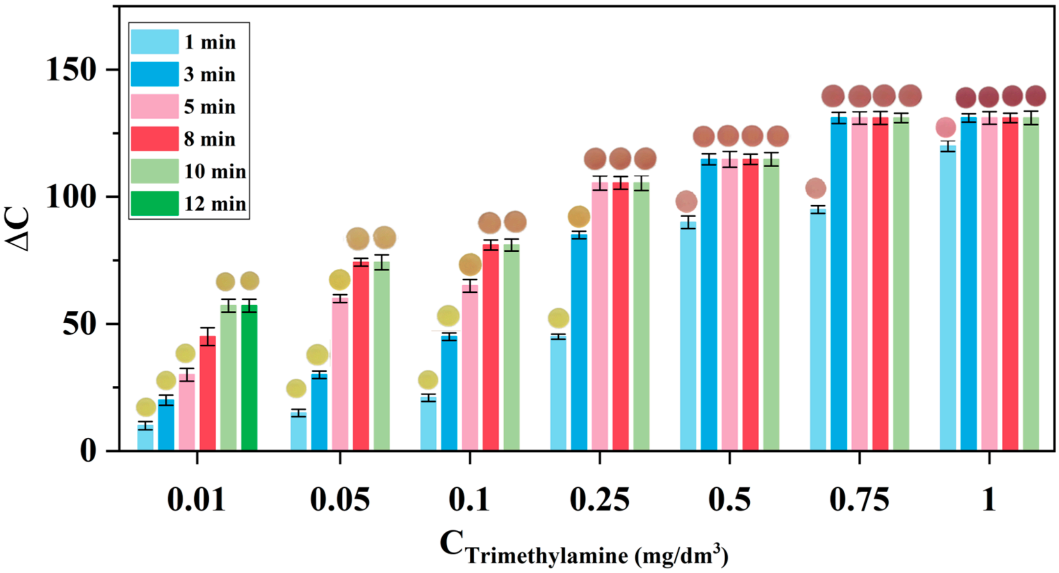

Figure S7. The ΔC values and images of Jug@AG hydrogel at different response times when sensing trimethylamine with concentrations of 0.01 mg/dm^3 -1 mg/dm^3 .

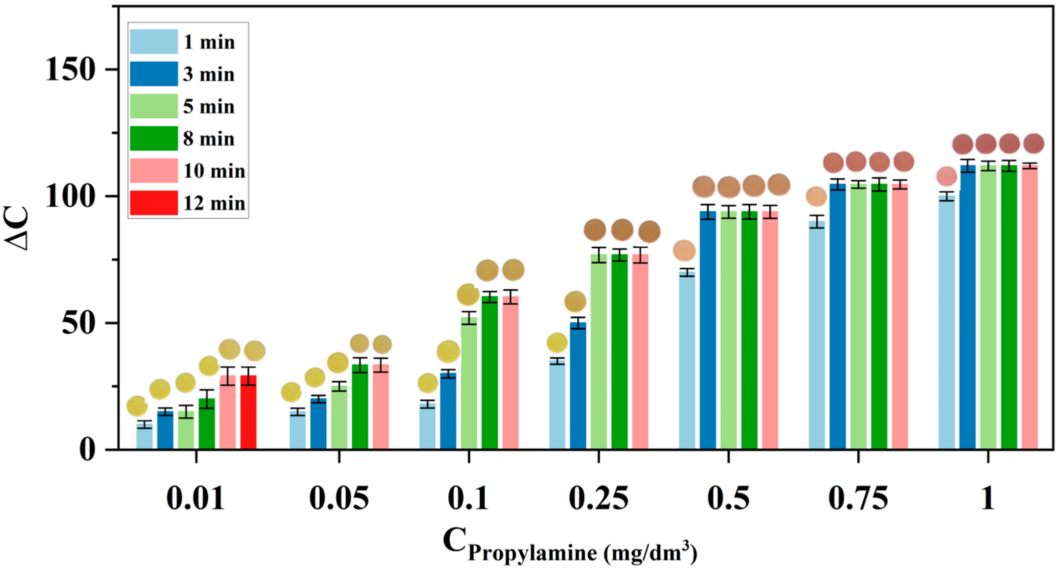

Figure S8. The ΔC values and images of Jug@AG hydrogel at different response times when sensing propylamine with concentrations of 0.01 mg/dm^3 -1 mg/dm^3 .

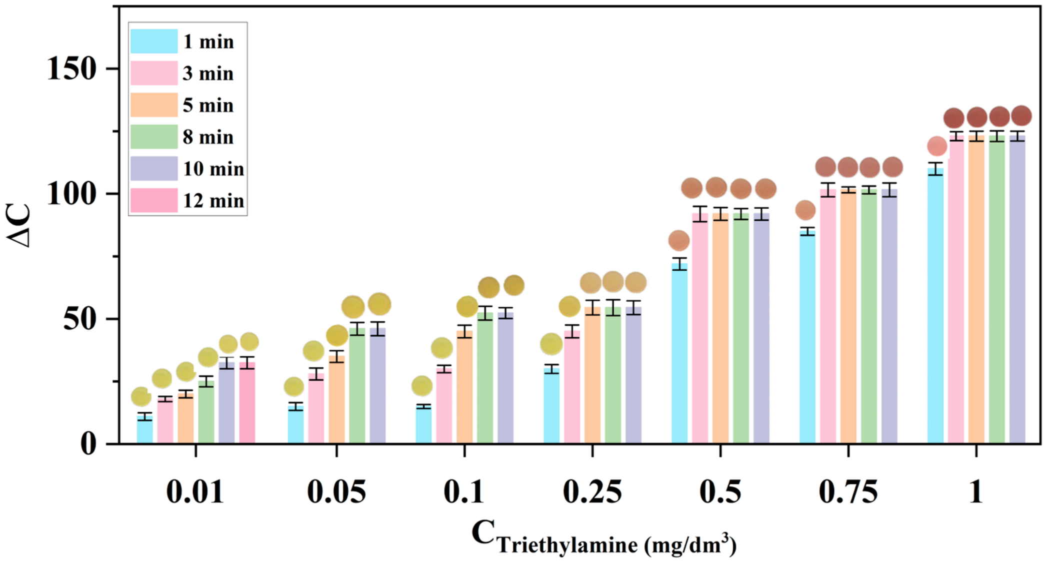

Figure S9. The ΔC values and images of Jug@AG hydrogel at different response times when sensing triethylamine with concentrations of 0.01 mg/dm^3 -1 mg/dm^3 .

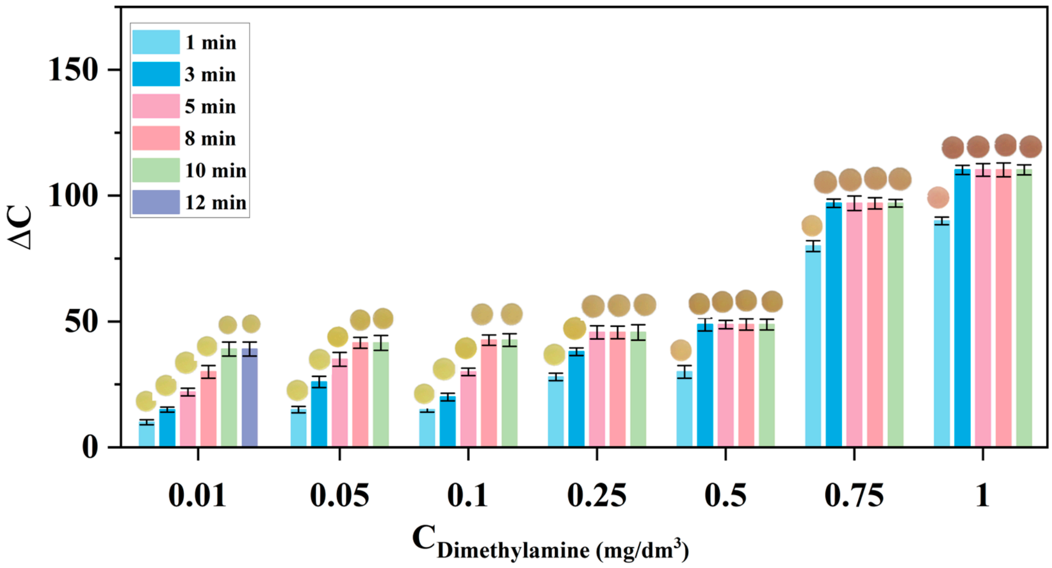

Figure S10. The ΔC values and images of Jug@AG hydrogel at different response times when sensing of dimethylamine with concentrations of 0.01 mg/dm^3 -1 mg/dm^3 .

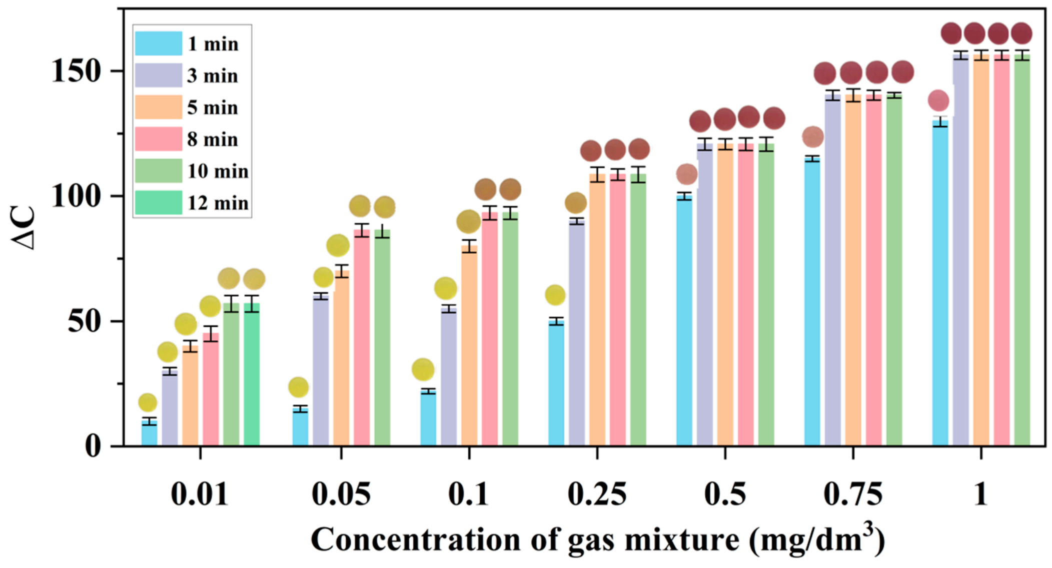

Figure S11. The ΔC values and images of Jug@AG hydrogel at different response times when sensing mixed gases with concentrations of 0.01 mg/dm^3 -1 mg/dm^3 .

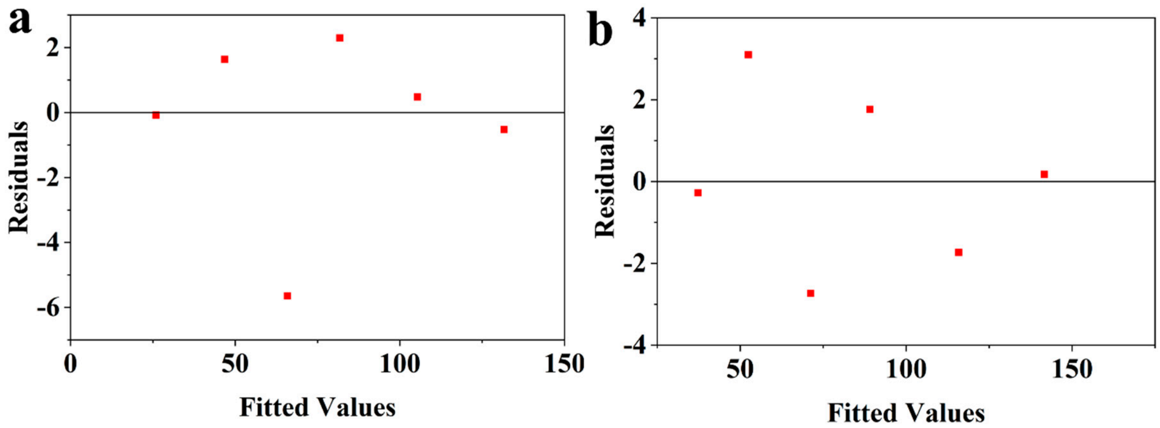

Figure S12. Residual analysis plots of (a) spiked pork and (b) spiked fish samples with the simulated TVB-N addition amount in the range of 5 mg/100 g-50 mg/100 g for detection.

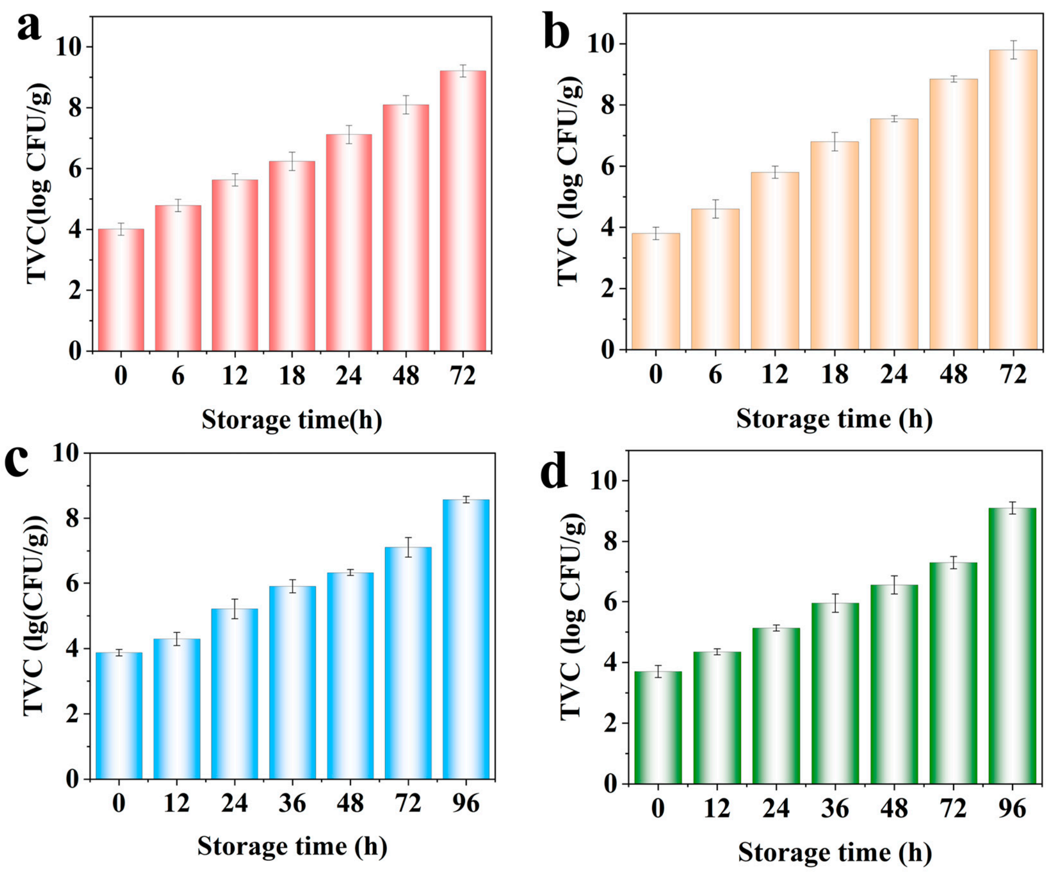

Figure S13. The variation of TVC of the sample with storage time at different temperatures: (a) pork and (b) fish stored at room temperature (25°C); (c) pork and (d) fish stored under cold storage (4°C).
